# Functional connectivity during frustration: a preliminary study of predictive modeling of irritability in youth

**DOI:** 10.1038/s41386-020-00954-8

**Published:** 2021-01-21

**Authors:** Dustin Scheinost, Javid Dadashkarimi, Emily S. Finn, Caroline G. Wambach, Caroline MacGillivray, Alexandra L. Roule, Tara A. Niendam, Daniel S. Pine, Melissa A. Brotman, Ellen Leibenluft, Wan-Ling Tseng

**Affiliations:** 1 Department of Radiology and Biomedical Imaging, Yale School of Medicine, New Haven, CT USA; 2 Department of Computer Science, Yale University, New Haven, CT USA; 3 Section on Functional Imaging Methods, Laboratory of Brain and Cognition, National Institute of Mental Health, Bethesda, MD USA; 4 Emotion and Development Branch, National Institute of Mental Health, Bethesda, MD USA; 5 Department of Psychiatry and Behavioral Sciences, University of California, Davis, Sacramento, CA USA; 6 Yale Child Study Center, Yale School of Medicine, Yale University, New Haven, CT USA; 7 Present Address: School of Medicine, Georgetown University, Washington, DC USA; 8 Present Address: School of Medicine, Temple University, Philadelphia, PA USA; 9 Present Address: Department of Psychology, The Pennsylvania State University, Philadelphia, PA USA

**Keywords:** Predictive markers, Reward

## Abstract

Irritability cuts across many pediatric disorders and is a common presenting complaint in child psychiatry; however, its neural mechanisms remain unclear. One core pathophysiological deficit of irritability is aberrant responses to frustrative nonreward. Here, we conducted a preliminary fMRI study to examine the ability of functional connectivity during frustrative nonreward to predict irritability in a transdiagnostic sample. This study included 69 youths (mean age = 14.55 years) with varying levels of irritability across diagnostic groups: disruptive mood dysregulation disorder (*n* = 20), attention-deficit/hyperactivity disorder (*n* = 14), anxiety disorder (*n* = 12), and controls (*n* = 23). During fMRI, participants completed a frustrating cognitive flexibility task. Frustration was evoked by manipulating task difficulty such that, on trials requiring cognitive flexibility, “frustration” blocks had a 50% error rate and some rigged feedback, while “nonfrustration” blocks had a 10% error rate. Frustration and nonfrustration blocks were randomly interspersed. Child and parent reports of the affective reactivity index were used as dimensional measures of irritability. Connectome-based predictive modeling, a machine learning approach, with tenfold cross-validation was conducted to identify networks predicting irritability. Connectivity during frustration (but not nonfrustration) blocks predicted child-reported irritability (ρ = 0.24, root mean square error = 2.02, *p* = 0.03, permutation testing, 1000 iterations, one-tailed). Results were adjusted for age, sex, medications, motion, ADHD, and anxiety symptoms. The predictive networks of irritability were primarily within motor-sensory networks; among motor-sensory, subcortical, and salience networks; and between these networks and frontoparietal and medial frontal networks. This study provides preliminary evidence that individual differences in irritability may be associated with functional connectivity during frustration, a phenotype-relevant state.

## Introduction

Irritability, defined as increased proneness to anger and frustration compared to peers [1], is a common presenting problem in child psychiatry [2]. Severe irritability in youth causes significant impairment in multiple domains and high rates of service use, hospitalization, and school suspensions [3]. Longitudinally, childhood irritability predicts adult anxiety and depressive disorders [4–6] and suicidality [7]. The highly impairing nature and long-lasting adverse outcomes of childhood irritability underscore a critical need to identify its etiology and pathophysiology, which remain unclear due to limited research. A better understanding of the brain mechanisms underlying irritability could facilitate the development of novel, neurobiologically informed treatments.

A recent translational model of irritability posits that a core pathophysiological deficit of irritability is aberrant responses to frustrative nonreward mediated by amygdala–frontostriatal dysfunction [1]. Frustrative nonreward, defined as the psychological state induced when an expected reward is withheld [8], is a construct in the negative valence systems within the research domain criteria matrix. In animals and humans, frustrative nonreward is associated with increased motor activity, aggression, and approach behavior [9, 10]. Neuroimaging paradigms that induce frustration in youth in a laboratory setting thus support a translational neuroscience approach that extends clinical observations on the central role of aberrant responses to frustration in irritability.

Using a frustration paradigm, a recent functional magnetic resonance imaging (fMRI) study in a large transdiagnostic sample with varying degrees of irritability found that, following frustration, higher levels of irritability (i.e., an average of parent and child reports) were related to greater frontal-striatal activation in regions including the dorsolateral prefrontal cortex, anterior cingulate cortex (ACC), inferior frontal gyrus (IFG), and caudate [11]. A few other studies using frustration paradigms in youth with irritability also report neural dysfunction in the same regions along with the amygdala and parietal cortex [12–14]. Much less is known about functional connectivity during frustration and its association with irritability. Functional connectivity may provide information about brain organization that is unique and complementary to regional activation, thus further facilitating discovery of brain–behavior associations [15].

Here, we examined the ability of functional connectivity across multiple networks in the brain to predict irritability using a machine learning approach, i.e., connectome-based predictive modeling (CPM) [16]. As a machine learning approach, CPM has built-in k-fold cross-validation that limits overfitting and increases generalizability of the findings [16]. CPM uses whole-brain functional connections and their associations with observed behaviors to build predictive models from this functional connectivity information and predict behavioral scores in novel subjects. CPM has been applied to psychological and psychiatric research to predict attention [17], opioid and cocaine use [18], and attention-deficit/hyperactivity disorder (ADHD) and autism symptoms [19]. This is the first study to use CPM to identify whole-brain patterns of disrupted networks underlying irritability using a frustrative nonreward paradigm.

The limited studies that examined functional connectivity during frustration reported decreased connectivity between IFG and periaqueductal gray extending to culmen in irritable youth [11] and increased connectivity between amygdala and ACC in healthy children and adults [20]. Other task-based data (without a frustration component) in irritable children showed that, during processing of social threat, higher irritability (plus anxiety) were related to decreased amygdala-medial prefrontal cortex (mPFC) connectivity [21]. During reward processing, especially omission of rewards, higher early childhood irritability was associated with altered amygdala–frontostriatal circuitry [22]. A few more studies used resting state, rather than task-based data, to reveal associations between functional connectivity and irritability. For example, children with severe temper outbursts, relative to healthy children or children with ADHD without temper outbursts, exhibited reduced resting state connectivity within ACC and increased connectivity between the mid-ACC and precuneus [23]. However, in a study with youths with severe mood dysregulation (the majority of whom met criteria for disruptive mood dysregulation disorder [DMDD]), amygdala-based resting connectivity did not differ between those with severe irritability and healthy controls [24].

Although these past studies provide preliminary evidence for neural connectivity abnormalities associated with irritability, all have utilized a seed-based approach by investigating amygdala-, ACC-, or IFG-based connectivity [11, 21, 23, 24]. Little is known about the patterns of disrupted networks underlying irritability across multiple networks in the brain. Moreover, only two studies examined functional connectivity using tasks probing frustrative nonreward [11, 20], a construct central to irritability [1]. It has been argued that tasks that tax individuals along a phenotype-relevant function, analogous to a cardiac stress test that identifies symptoms not observable at rest, may amplify individual differences in neural circuitry mediating the particular phenotype and thus improve the detection of brain–behavior associations [25, 26]. As such, it is important to test whether tasks involving frustrative nonreward are better suited to elicit individual differences in network abnormalities associated with irritability, compared to resting state or other tasks without a frustration component. In this study, we utilize a modified change-signal task [27] with rigged feedback to probe cognitive flexibility (i.e., the ability to adapt one’s thinking and behavior in response to changing environmental conditions/demands) [28] under frustrative nonreward. This task is novel and unique, relative to previous tasks probing frustrative nonreward [11, 20], in the following aspects: (a) it probes both motor inhibition and cognitive flexibility, two processes critical for emotion regulation, whereas previous studies probed attention orienting [11] and inhibitory control [20] in separate lines of work and (b) the frustration and nonfrustration blocks are interspersed randomly, whereas the order was fixed in previous tasks [11, 20].

This preliminary study aimed to address the aforementioned gaps in the literature by examining functional connectivity for multiple networks across the whole brain, rather than a single seed-defined functional network, during frustrating vs. nonfrustrating states using CPM [16]. Specifically, we tested the ability of functional connectivity to predict irritability symptoms in a transdiagnostic sample of 69 youths with varying levels of irritability. We used CPM to associate connectivity with dimensional measures of irritability, rather than categories, at a subject-by-subject level to maximize the information available to characterize individual differences in brain–behavior relations. Given the past literature, we hypothesized that functional connectivity during a frustrating, but not nonfrustrating, state would be associated with individual differences in irritability and that networks involving the amygdala, striatum, ACC, and prefrontal cortex would be the most predictive of irritability.

## Materials and methods

### Participants

This study included 69 youths with varying levels of irritability across four diagnostic groups: 20 DMDD, 14 ADHD, 12 anxiety disorder, and 23 healthy controls with no history of DSM-5 diagnosis (see Table 1 for sample characteristics). Participants were recruited by the National Institute of Mental Health (NIMH) Intramural Research Program. This study was approved by the NIMH IRB, and written consent/assent was obtained from parents/children. See Supplementary Information for detailed diagnostic/clinical assessments and exclusion criteria.

**Table 1:** Sample characteristics.

	*n* (%) or mean (SD)	Range
Age, mean (SD), years	14.55 (2.85)	8–22
Gender, *n* (%)^a^	38 (55.10)	─
IQ, mean (SD)^b^	111.75 (11.45)	87–133
SES, mean (SD)^c^	38.34 (19.99)	20–114
Motion, mean (SD)^d^	0.07 (0.03)	0.02–0.16
Irritability measures, mean (SD)		
Child-reported ARI	1.79 (2.04)	0–9
Parent-reported ARI^e^	2.80 (3.35)	0–10
Primary diagnosis, *n* (%)		
DMDD	20 (28.99)	─
ADHD	14 (20.29)	─
Anxiety	12 (17.39)	─
No diagnosis	23 (33.33)	─

*ADHD* attention-deficit/hyperactivity disorder, *ARI* affective reactivity index, *DMDD* disruptive mood dysregulation disorder, *SES* socioeconomic status.

^a^Coded as 0 (male) and 1 (female); *n* (%) is for the male.

^b^Measured by the Wechsler abbreviated scale of intelligence. Missing data for one participant.

^c^Measured by the Hollingshead two-factor index. Missing data for eight participants.

^d^Calculated as the mean Euclidean distance of framewise volume shift after censoring.

^e^Missing data for five participants.

### Measures

Irritability was measured using the parent report and child report of the Affective Reactivity Index (ARI) scale [29]. The ARI is a six-item short scale assessing the frequency, duration, and threshold of irritability [29], providing a measure of trait irritability. It has good internal consistency (*α* ≥ 0.80), discriminant validity, and test–retest reliability [29, 30]. The internal consistency in this sample is 0.92 for the parent report and 0.79 for the child report. Items were rated on a 0–2 scale and yielded a total score of 0–12. For >95% of the sample, these measures were collected within 1 month of scanning (>65% collected within a week of scanning).

### fMRI task

**Fig. 1:**
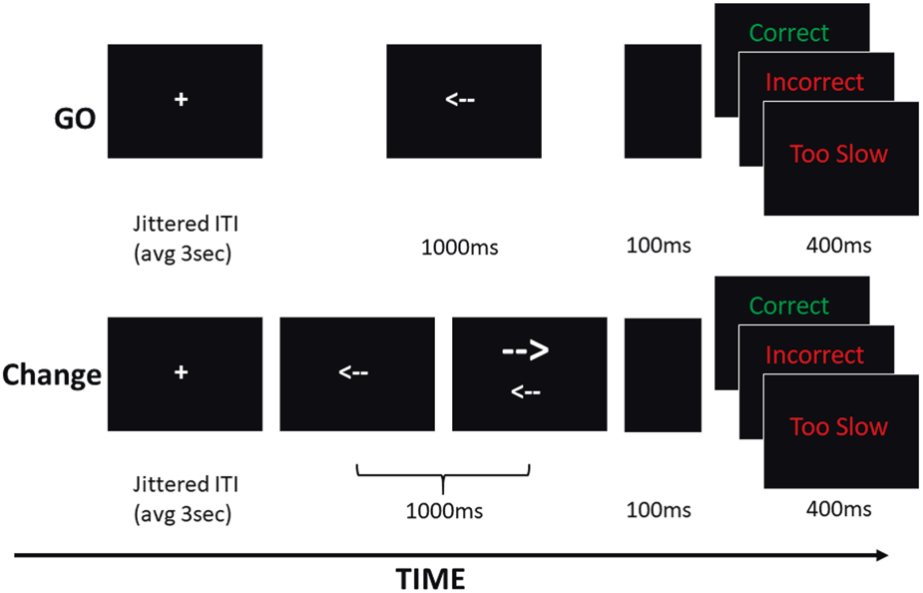
Trial timing and structure during the modified change-signal task. ITI intertrial interval.

Participants completed a modified change-signal task [27] during fMRI data collection. The task consisted of two trial types: go (60%) and change (40%). See Fig. 1 for the trial timing and structure. The task was divided into six functional runs (each lasted ~6 min) and consisted of two blocks: a “frustration” block (50% error rate on change trials plus 20% rigged feedback on correct go trials) and a “nonfrustration” block (10% error rate on change trials), the order of which was randomized within run. This task thus evokes frustrative nonreward (i.e., the psychological state induced when an expected reward is withheld) [8] via manipulation of the error rate and inclusion of rigged feedback. Of note, frustrative nonreward is closely related to negative prediction error, i.e., when a reward is worse or less than predicted [31]. Given our focus on “psychological/brain state” under frustration, we use the term frustrative nonreward, rather than negative prediction error, throughout the paper. The scan length of 36 min provides a good amount of data to estimate functional connectivity [32]. At the end of each block, participants self-reported their feelings of frustration using a nine-point Likert scale, providing a measure of state irritability. Overall, participants reported feeling more frustrated during frustration than nonfrustration blocks, supporting the task’s validity as a frustrative nonreward paradigm, which did not vary as a function of age. See Supplementary Information for task details and behavioral analyses/results.

### Data acquisition and preprocessing

Neuroimaging data were acquired on a 3-T General Electric scanner using a 32-channel head coil. Data were preprocessed using the Analysis of Functional NeuroImages with standard preprocessing procedures (e.g., despiking, slice timing correction, coregistration). See Supplementary Information for details regarding scan parameters, preprocessing, and head motion. Several covariates of no interest were regressed out from the data including the 12 motion parameters (six rigid body motion parameters and six temporal derivatives), mean white matter signal, mean cerebrospinal fluid signal, mean global signal, and the linear, quadratic, and cubic drifts. Global signal regression was performed as it strengthens the association between functional connectivity and behavior, leading to better performing and generalizing predictive models [33].

### Connectivity matrices

Whole-brain functional connectivity was assessed as described previously [25, 26]. Briefly, network nodes were defined using the Shen 268-node functional brain atlas that includes the cortex, subcortex, and cerebellum [34, 35]. 1The atlas was warped from MNI space into single-subject space. Task connectivity was calculated on the basis of the “raw” task time courses, without removal of task-evoked activity, as validated in previous studies [25, 26]. In addition, as the task blocks are long (~3 min), this approach is similar to the one used in [17] and approximates a continuous performance task. We have previously shown that calculating connectivity matrices in this manner emphasizes individual differences in connectivity [25] and increases CPM performance [26]. For every node, a mean time course was calculated by averaging the time courses of all of its constituent voxels. Pairwise correlations were computed between all pairs of nodes, and Pearson correlation coefficients were Fisher *z*-transformed to yield symmetric 268 × 268 connectivity matrices, as is standard in the field.

### Connectome-based predictive modeling

CPM was conducted to predict child- and parent-reported ARI scores using previously validated custom MATLAB scripts [16]. 2CPM uses connectivity matrices and phenotypic data from individuals as input to generate a predictive model of the behavioral data from connectivity matrices. Positive networks are networks for which increased edge weights (increased connectivity) are associated with the variable of interest, and negative networks are those for which decreased edge weights (decreased connectivity) are associated with the variable of interest. See Supplementary Information for details and a schematic of CPM (Fig. S1). One hundred iterations of tenfold cross-validation were used to train and evaluate models.

### Localization of predictive networks

Predictive networks identified using CPM are complex and composed of multiple brain regions and networks. Similar to previous CPM studies, predictive networks were summarized at multiple levels of data reduction including at the edge, node, and network level [36]. Macroscale brain regions (e.g., prefrontal cortex, cerebellum) and canonical functional network localizations (e.g., frontoparietal, motor sensory) based on previous work [35] were presented. In addition, for each node, the network theory measure degree was calculated as the sum of the number of edges for each node that belonged to the predictive networks. Visualizations of predictive edges were created using BioImage Suite Web. 3See details in Supplementary Information.

### Statistical analysis

The correspondence between predicted and actual values, or model performance, was assessed using Spearman’s rank correlation (ρ; to avoid strong distribution assumptions) and root mean square error (defined as: $\mathrm{RMSE}(\mathrm{predicted},\mathrm{actual}) = \surd ( {1/n\sum_{\{ {i = 1} \}}^n ( {\mathrm{actual}_i - \mathrm{predicted}_i} )^2})$). Model performance of the median performing model from the 100 iterations of tenfold cross-validation is reported. When using cross-validation, analyses in the left-out folds are not independent, and the number of degrees of freedom is thus overestimated for parametric *p* values. Instead of parametric testing, we therefore performed permutation testing. To generate null distributions for significance testing, we randomly shuffled the correspondence between behavioral variables and connectivity matrices 1000 times and re-ran the CPM analysis with the shuffled data. Based on these null distributions, the *p* values for predictions were calculated as: $p = (\# \{ \rho _{\mathrm{null}} > \rho _{\mathrm{median}}\} + 1)/1001$, where #{ρ_null_ > ρ_median_} indicates the number of permutated predictions numerically greater than the median of the unpermutated predictions. As we expect a positive association between predicted and actual values, one-tailed *p* values are reported. Comparison between models was performed using Steiger’s test to compare dependent correlation coefficients.

## Results

### Prediction of trait irritability

**Fig. 2:**
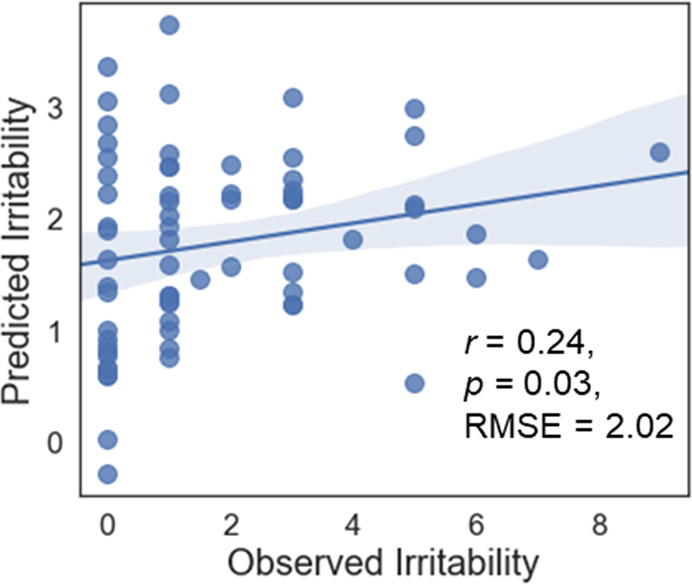
Correlation between observed (*x*-axis) and predicted (*y*-axis) irritability generated using CPM. RMSE root mean square error. Shaded area represents 95% confidence interval.

The overall CPM model successfully predicted child-reported ARI using functional connectivity from the frustration blocks (ρ = 0.24, RMSE = 2.02, *p* = 0.03, permutation testing, 1000 iterations, one-tailed; Fig. 2). Given the confounding effect of motion on functional connectivity, the wide age range of the sample, potential sex differences, the co-occurrence of ADHD and anxiety symptoms with irritability, and potential medication confound, we tested whether our findings remained after adjusting for the effects of motion, age, sex, ADHD and anxiety symptoms, and medications. Follow-up comparisons adjusting for head motion (ρ = 0.24, RMSE = 1.98, *p* = 0.03), age (ρ = 0.22, RMSE = 2.01, *p* = 0.04), sex (ρ = 0.24, RMSE = 2.0, *p* = 0.04), ADHD symptoms (ρ = 0.22, RMSE = 2.03, *p* = 0.04), anxiety symptoms (ρ = 0.22, RMSE = 2.03, *p* = 0.04), stimulants (ρ = 0.24, RMSE = 2.01, *p* = 0.03), nonstimulants ADHD medications (ρ = 0.24, RMSE = 2.01, *p* = 0.03), antidepressants (ρ = 0.25, RMSE = 2.01, *p* = 0.01), and antipsychotics (ρ = 0.23, RMSE = 2.01, *p* = 0.04) demonstrated similar prediction performances. When the CPM model was divided into independent positive and negative predictive networks, only the positive predictive network contributed to prediction (positive network: ρ = 0.29; negative network: ρ = 0.04). We were not able to predict child-reported ARI using functional connectivity from either the nonfrustration blocks (ρ = 0.06, RMSE = 2.17, *p* = 0.26) or the difference between the frustration and nonfrustration connectomes (ρ = −0.05, RMSE = 3.83, *p* = 0.49). These prediction performances were significantly worse than that using the frustration blocks (*z* = 2.67, *p* = 0.008; *z* = 2.82, *p* = 0.005). Finally, we were not able to predict parent-reported ARI with CPM using functional connectivity from the frustration blocks (ρ = −0.07, RMSE = 3.48, *p* = 0.61), nonfrustration blocks (ρ = −0.04, RMSE = 3.43, *p* = 0.59), or the difference between the connectomes (ρ = 0.03, RMSE = 3.72, *p* = 0.52). These predictions were significantly worse than the predictions of child-reported ARI using the frustration blocks (*z* = 2.5, *p* = 0.01; *z* = 2.3, *p* = 0.02; *z* = 2.0, *p* = 0.05).

Given the high co-occurrence between irritability and ADHD symptoms, we conducted additional analysis to further disentangle the relationship between irritability and ADHD and found that the networks predicting ADHD were largely independent of those predicting irritability (see Supplementary Information).

### Anatomical and network localization of circuits predicting child-reported trait irritability

**Fig. 3:**
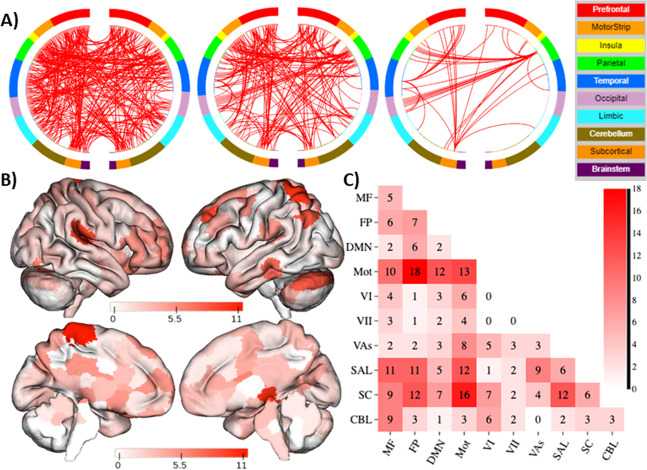
CPM predicts irritability. **A** Edges that contributed to the CPM model organized by macroscopic brain regions. To help visualizing these complex networks, edges only belonging to nodes with five or more edges (degree ≥ 5; middle) and 10 or more edges (degree ≥ 10; right) are also shown. **B** Visualization of node degree (i.e., the sum of predictive edges for a node for the positive networks). Darker color indicates higher degree. **C** Within and between network connectivity for the positive network. Cells represent the total number of edges connecting nodes within and between each network, with darker colors indicating a greater number of edges. As the negative network did not contribute to prediction, only the positive network is shown in all visualizations. Visualization created using BioImage Suite Web, http://bisweb.yale.edu/. MF medial frontal, FP frontoparietal, DMN default mode network, Mot motor/sensory, VI visual A, VII visual B, VAs visual association, SAL salience, SC subcortical, CBL cerebellum.

Because the negative network did not contribute to prediction, we only localized the positive network. A total of 266 edges positively predicted child-reported ARI, consisting of 56 ipsilateral connections in the right hemisphere, 82 ipsilateral connections in the left hemisphere, and 128 connections between the right and left hemispheres (Fig. 3A). These connections included equal number of long- and short-range connections (45% long range; 55% short range, *χ*^2^ = 0.66, *p* = 0.42). In addition, 214 brain regions (out of 268) and all 10 canonical networks contributed to this model. Nodes with the greatest number of edges were primarily located in the left cerebellum, left parietal lobe, right thalamus, and motor cortex (Fig. 3B). At the network level, connections within the motor-sensory network and between the motor-sensory, subcortical, and salience networks, and between these networks and the medial frontal and frontoparietal networks contributed the majority of edges to the positive network (Fig. 3C).

### Prediction of state irritability

We investigated whether CPM could predict state irritability, i.e., participants’ self-rated frustration during task. Neither the original CPM model (ρ = 0.16, RMSE = 1.97, *p* = 0.14, permutation testing, 1000 iterations, one-tailed) nor models trained from the frustration (ρ = 0.18, RMSE = 2.17, *p* = 0.12, permutation testing, 1000 iterations, one-tailed) or nonfrustration blocks (ρ = 0.19, RMSE = 1.77, *p* = 0.11, permutation testing, 1000 iterations, one-tailed) significantly predicted state irritability.

### Prediction of trait irritability using task activation

Given that task connectivity predicted child-reported irritability, we also examined if task activation could predict child-reported irritability (Supplementary Information). In brief, we found limited evidence that task activation predicted child-reported irritability.

## Discussion

This is the first study to apply predictive modeling (i.e., CPM) to identify whole-brain patterns of disrupted networks underlying irritability using a frustrative nonreward paradigm. We used CPM as a machine learning approach to associate connectivity with dimensional measures of irritability, rather than categories, at a subject-by-subject level to maximize the information available to characterize individual differences in brain–behavior relations. Given the limited sample size (*n* = 69), our results should be treated as preliminary. Nonetheless, our CPM approach showed that functional connectivity during frustration (but not nonfrustration) blocks was associated with child-reported irritability. The predictive networks of child-reported irritability were primarily within the motor-sensory network; among the motor-sensory, salience, and subcortical (including amygdala, thalamus, striatum) networks; and between these networks and the frontoparietal and medial frontal networks. Results were adjusted for age, sex, motion, medications, ADHD, and anxiety symptoms.

Functional connectivity is commonly examined using resting state scans. In this study, we found that functional connectivity during a “frustrated” state was more predictive of youths’ trait-level irritability than functional connectivity during a “nonfrustrated” state. Thus, the frustration task may serve as a “mental stress test” [25, 26] to evoke individual differences in connectivity related to irritability. That is, researchers may be more likely to observe disrupted connectivity and brain dysfunction in youth with irritability when the brain system is “stressed” by frustration than when they are “at rest.” These results align with other CPM studies using task-based connectivity. For example, task-based connectivity using salient infant faces and cry stimuli was associated with caregiving measure of early postpartum mothers [37], and connectivity from relevant tasks were associated with relapse to cocaine and opioids in polysubstance using individuals [18]. Overall, our findings support the importance of examining functional connectivity during a phenotype-relevant state such as frustration to better probe individual differences in brain–behavior associations and thus facilitate biomarker discovery [38].

Past research employed a seed-based approach focused on amygdala-, ACC-, or IFG-based connectivity and linked irritability and frustration to neutral circuits connecting amygdala, ACC, mPFC, and striatum [20–23]. Importantly, the current study extends prior literature by examining connectivity of multiple functional networks across the brain. Indeed, a much larger networks were implicated in irritability than previously found [20–23]. Most of the networks predicting irritability were within the motor-sensory network and between motor-sensory, salience (e.g., ACC), and subcortical (e.g., amygdala, striatum) networks as well as between these three networks and medial frontal (e.g., mPFC) and frontoparietal (e.g., IFG) networks. Our finding involving the salience, subcortical, medial frontal, and frontoparietal networks was consistent with prior seed-based literature linking IFG, amygdala, ACC, mPFC, and striatum [20–23]. One novel finding of this study is the contribution of within motor-sensory network, and between motor-sensory and frontoparietal, subcortical, salience, and medial frontal networks to the prediction of irritability.

A recent fMRI study of irritability in youth used a stop-signal task [39], which is similar to our “change-signal” task except that only ours requires the execution of an alternate response after inhibiting the prepotent response. This study found that irritability in youth is associated with decreased activation in the primary motor and somatosensory cortex [39]. Research on the irritability-related construct of aggression also finds links between aggression and dysfunction in the primary motor cortex [40, 41]. Moreover, structural connectivity and voxel-based studies suggest that irritability in youth is associated with abnormal white matter microstructure in the corticospinal tract, a major motor pathway [42], and lower gray matter volume in the primary motor and somatosensory cortex [39] and presupplementary motor area [43]. Behaviorally, research in animals and human suggest that following frustration, animals and human showed increased motor activity, aggression, and approach behavior [9, 10]. These are consistent with our finding that connectivity within the motor-sensory network and between motor-sensory and other networks (e.g., frontoparietal, subcortical, salience, and medial frontal) are among the main predictive networks of irritability. Dysfunction in the mPFC, parietal lobe, and regions in the subcortical (amygdala, striatum) and salience (ACC) networks have been reported in youth with irritability, especially when frustrated [11–14]. Increased connectivity between the motor-sensory network and other networks (including the frontoparietal, subcortical, salience, and medial frontal networks) during frustration in youth with irritability may mediate dysregulated attention and motor control and disrupt integration of motor-sensory, emotional, and cognitive information during frustration in youth with irritability. As irritable youths become frustrated, they may activate the motor network and its connection to other networks to a greater extent than nonirritable youths, thus resulting in the increased motor activity that characterizes the temper outbursts and aggressive behavior associated with irritability [1].

In contrast, functional connectivity during the task was not associated with parent-reported irritability. This is surprising given previous studies linking brain function [44] and connectivity [22] to parent reports of irritability. Notably, our sample included youths who were significantly older (mean age = 14.55 years; four subjects aged 18–22 years) than previous studies (mean age = 4.27 years [22] and 13.06 years [44]). It is well documented that parent–child informant disagreement tends to be greater for internalizing and mood symptoms, which are less observable to others, than externalizing symptoms [45], hence an emphasis on children’s self-reports of internalizing symptoms [46, 47]. Perhaps when youths get older, they become better and more accurate informants of their own irritability than their parents, thus enabling detection of the brain–behavior association.

Functional connectivity was not associated with state irritability (i.e., subjects’ moment-to-moment frustration ratings during task), despite participants’ feelings of greater frustration during frustration than nonfrustration blocks. This may be a type 2 error or due to the generally poor reliability of task measures [48]. Objective measures such as physiological data (e.g., skin conductance and heart rate) may provide an additional probe of state irritability. It remains to be determined if disrupted functional connectivity during frustration better reflects individual differences in trait irritability than in state irritability. Given the low convergent validity between self-report and task measures and the fact that each may assess different aspects of the same cognitive process or behavioral tendency [49], future work should continue to include both types of measurements as probes for individual differences in irritability.

When we used task activation from five main events to predict irritability using common, standard machine learning approaches (i.e., SVR, LASSO, ridge, and elastic net), we found limited evidence that task activation predicted irritability. Only the correct change trials during frustration blocks significantly predicted child-reported irritability when using elastic net with α = 0.50 specifically. This may be due to the low reliability of regional activation, relative to functional connectivity [50], and is consistent with the increasingly recognized notion that psychiatric disorders and phenotypes are more likely to involve complex alterations in neural network and connectivity rather than localized abnormal activation within specific regions [51]. However, future fMRI research should continue to investigate both regional activation and connectivity patterns, as each provides important information to advance the understanding of the neural mechanisms mediating phenotypes of interest [15].

A common concern about predictive modeling in human neuroscience is that resulting models often seem to explain disappointingly little variance in the predicted measure, particularly when compared to results derived from explanatory models [52]. In contrast to explanatory models that use all available data to generate a model, predictive models attempt to prevent overfitting by validating the model through cross-validation with strict separation of the training and testing data. Thus, correlations reported from predictive modeling are typically lower than those from explanatory models but are much closer to the true underlying effect sizes. Our reported effect sizes align with most predictive modeling studies [53] and with recent mega-analyses comprised of thousands of subjects [54].

While this study provides novel contribution to the understanding of neural networks implicated in irritability, several limitations should be noted. While our sample is moderate for an fMRI study, it is relatively small for predictive models. We used tenfold cross-validation to increase our generalizability over explanatory models, consistent with current best practices for predictive modeling [52, 55]. Nonetheless, the use of predictive modeling alone does not guarantee generalizability to another sample. Thus, our results remain preliminary, and replication of our findings is needed using large, diverse samples. Relatedly, our findings may apply only to the disorders sampled here (DMDD, anxiety disorder, and ADHD) and not to other disorders (e.g., depression, autism, and bipolar disorder) in which irritability is also common. Of note, failure to generalize may reflect inherent differences in samples. The underlying predictive features may be different for different populations and reflect important group differences in underlying neural circuitry. In addition, the sample had a wide age range, and the limited sample size precluded us from stratifying our analysis by age groups. However, our results are unlikely to be driven by the wide age range, as we found that (a) the validity of our task as a frustrative nonreward paradigm did not vary as a function of age; (b) irritability was not related to age; and (c) our analyses covaried for age. Still, future research with a larger sample or a narrower age range is warranted to determine if our findings can be generalized across age groups. Another interesting future direction would be to test the generalizability of our CPM results to frustrative nonreward tasks probing different processes other than cognitive flexibility.

In conclusion, in this pilot work, we found that functional connectivity when youths were frustrated was associated with their self-reported irritability symptoms outside of the scanner. Neural networks supporting motor function, action preparation, attention control, and integration of motor-sensory, emotional, and cognitive information are among the most predictive networks of irritability. Future research with large samples is necessary to replicate these findings.

## Funding and disclosure

This study was supported by the Intramural Research Program of the NIMH, and it was conducted under projects ZIA-MH002778 (clinical protocol NCT00006177), ZIA-MH-002781 (NCT00018057), and ZIA44 MH002786 (NCT00025935). W-LT is supported by research grant from the NIMH (R00MH110570). The authors reported no biomedical financial interests or potential conflicts of interest.

## Supplementary information

Supplementary Online Content for Scheinost et al.: Functional Connectivity During Frustration: A Preliminary Study of Predictive Modeling of Irritability in Youth

## Footnotes

1 https://www.nitrc.org/projects/bioimagesuite/

2 https://github.com/YaleMRRC/CPM

3 https://bioimagesuiteweb.github.io/webapp/connviewer.html
