# Supplementary material for: Functional connectivity during frustration: a preliminary study of predictive modeling of irritability in youth
Source: Neuropsychopharmacology. 2021 Jan 21;46(7):1300–6. doi: 10.1038/s41386-020-00954-8 (PMC8134471; doi:10.1038/s41386-020-00954-8)
Supplement: Supplementary file 1 — Supplementary Online Content for Scheinost et al.: Functional Connectivity During Frustration: A Preliminary Study of Predictive Modeling of Irritability in Youth [file 41386_2020_954_MOESM1_ESM.docx]

**Supplementary Online Content for Scheinost et al.:**

Functional Connectivity During Frustration: A Preliminary Study of Predictive Modeling of Irritability in Youth

**Participant Assessment**

All children were assessed using the Kiddie Schedule for Affective Disorders and Schizophrenia for School-Age Children-Present and Lifetime version (K-SADS-PL) (1).1 The K-SADS-PL, including an additional module for assessing disruptive mood dysregulation disorder (DMDD; available on request), was administered separately to children and parents by masters- or doctoral-level clinicians with good inter-rater reliability (kappa ≥ 0.7 for all diagnoses). Clinicians were trained to levels of acceptable reliability by rating video recordings and conducting supervised assessments. Children in each clinical group met criteria for primary diagnoses of DMDD, attention-deficit/hyperactivity disorder (ADHD), and anxiety disorders (ANX). Children with ADHD and ANX did not meet criteria for DMDD, but youth with DMDD may have co-occurring ADHD and ANX. Patients with ANX met criteria for generalized anxiety disorder, separation anxiety disorder, and/or social phobia; the diagnosis of DMDD and major depressive disorder (MDD) were exclusionary for the ANX group. Diagnoses were confirmed in consensus conferences chaired by a senior psychiatrist (coauthors EL, MB, or DP). Exclusion criteria included IQ<70, pervasive developmental or neurological disorders, substance abuse within the past two months, or lifetime history of psychosis, conduct disorder, and unstable and/or chronic medical illness. Additional exclusion criteria for ANX patients were current Tourette’s syndrome, obsessive-compulsive disorder, major depressive disorder, post-traumatic stress disorder, current psychotropic medication use, or suicidal ideation. Psychotropic medication use was not exclusionary for youths with DMDD or ADHD. The most commonly used psychotropic medications were stimulants (75% DMDD; 71.4% ADHD), non-stimulant ADHD medications (15% DMDD; 7.1% ADHD), antidepressants (50% DMDD; 7.1% ADHD), and antipsychotics (35% DMDD; 0% ADHD).

The four diagnostic groups varied in age (*F*_3,65_=2.95, *p*=.039). This was driven by the DMDD group being older than the healthy controls and the ANX group – the mean age (in years) of the four groups were: 13.74 for healthy controls, 16.05 for DMDD, 14.34 for ADHD, and 13.86 for ANX. However, age was not associated with dimensionally-assessed irritability using the Affective Reactivity Index (ARI; *r*=-.11, *p*=.35 for child-reported ARI, *r*=.08, *p*=.52 for parent-reported ARI). Nonetheless, our connectome-based predictive modeling (CPM) analyses adjusted for age given the wide age range of the sample.

ADHD symptoms were measured by the ADHD-Index subscale of the Conners’ Parent Rating Scale-Revised: Long Form (CPRS-R: L) (2).9 It is a widely used rating scale for ADHD symptoms and has good reliability and validity (2).9 Age- and gender-referenced T-score was used in the analysis. The range of the ADHD-Index T-score in this sample was 40-83 (mean = 57).

Anxiety symptoms were measured by the Screen for Child Anxiety Related Emotional Disorders (SCARED), a validated, 41-item parent- and self-rated measure (3).8 The SCARED parent- and child-report measure is widely used in clinical and research settings with good psychometric properties including good internal consistency, measurement invariance, strong test-retest reliability, and adequate external validity (4). The averaged total score of parent- and child-reported SCARED was used in the analysis. The range of the scores was 0.5-43.5 (mean = 12.98).

**fMRI Task**

The modified Change-Signal task (5) probed cognitive flexibility under frustrative nonreward during fMRI data collection. The task consisted of two trial types: go and change. On go trials (60% of all trials), participants were presented with a go signal (“<--” or “-->”) and were instructed to press a button (left or right) to indicate the direction of the arrow. On change trials (40% of all trials), participants were presented with a go signal, followed by a “change” signal (a second, larger arrow above the first arrow) with some delay. Subjects were instructed to respond in the direction of the change signal, instead of the go signal. After participants responded, they were provided with feedback (“correct,” “incorrect,” and “too slow”). Thus, change trials required participants to inhibit a prepotent response (“go”) and to substitute an alternative response (“change”) for the prepotent one; thus, the task, particularly the change trials, require motor inhibition and cognitive flexibility for successful completion. On change trials, the delay between the go signal and the change signal started at 250ms and then increased or decreased by 50ms on the subsequent trial depending on the algorithm that determined the overall accuracy (see below).

Frustration was evoked by manipulating task difficulty i.e., changing timing of the delay (starting at 250ms) between the go and change signal (e.g., a shorter delay makes it easier to complete the change trial correctly and a longer delay makes it harder to complete the change trial correctly). “Frustration” blocks were programmed to have an error rate of 50% and “non-frustration” blocks were programmed to have an error rate of 10% on change trials. To ensure that the “frustration” blocks were sufficiently frustrating, we also provided participants rigged feedback (“too slow”) on 20% of the go trials when they performed accurately. At the end of each block, participants self-reported their feelings of frustration using a 9-point Likert scale, providing a measure of state irritability. Overall, participants reported feeling more frustrated during frustration than non-frustration blocks (Mean±SD=3.43±1.80 vs. 2.62±1.52, *p*<.001), supporting the task’s validity as a frustrative nonreward paradigm, which did not vary as a function of age (correlations between age and frustration ratings were *r*=-.14, *p*=.25 for the non-frustration blocks and *r*=-.09, *p*=.45 for the frustration blocks.

Each block (frustration and non-frustration) contained 30 trials (18 go trials and 12 change trials). All participants started the task with $50. They were instructed that they would lose $0.50 for each incorrect answer and that their job was to perform as accurately as possible and to try to keep as much money as possible. Monetary reward was not shown during feedback with each trial; it was shown at the end of each block as a running total.

**fMRI Acquisition, Preprocessing, and Head Motion**

A high-resolution anatomical scan (1-mm slices, 3-dimensional spoiled gradient-echo, 7° flip angle, minimum full echo time, 256x256 matrix, 25.6cm field of view) and gradient echo-planar imaging images were collected (repetition time [TR]=2300ms, echo time=30ms, 24cm field of view, voxel size=2.5,2.5,3 mm, 165 volumes per run, flip angle=70°).

The first four volumes of each functional run were discarded to allow for the magnetization to reach a steady state. TR pairs with a Euclidean norm motion derivative >0.3mm were censored during linear regression. The average number of censored TRs was 70 (fraction=.07), which was not correlated with either child- or parent-reported ARI (r’s<.03, p’s>.79). Finally, for each participant, all preprocessed runs were variance normalized and concatenated.

As head motion has been shown to confound connectivity studies, we calculated the average frame-to-frame displacement for each participant’s data. Only subjects with an average frame-to-frame displacement after censoring <0.25mm and TR censor fraction <.25 were included in this study. Thirteen subjects who did not meet these criteria and additional two subjects with missing child-reported ARI were excluded, resulting in a final sample of N=69. Neither motion during the frustration nor non-frustration blocks correlated with child- or parent-reported ARI (r’s<.11, p’s>.37), and there were no differences in the magnitude of these correlations between frustration and non-frustration blocks (z’s<0.90, p’s>.36). In addition, we adjusted for frame-to-frame displacement in our CPM analysis using partial correlation.

**Connectome-based predictive modeling (CPM)**

CPM uses connectivity matrices and phenotypic data from individuals as input to generate a predictive model of the behavioral data from connectivity matrices. See Figure S1 for a schematic of CPM. Edges and phenotypic data from the training data set are correlated using regression analyses with either Pearson’s correlation or partial correlation (when adjusting for possible confounds) to identify positive and negative predictive networks using an edge-selection threshold of p<.05. Positive networks are networks for which increased edge weights (increased connectivity) are associated with the variable of interest, and negative networks are those for which decreased edge weights (decreased connectivity) are associated with the variable of interest. Single-subject summary statistics are then created as the sum of the significant edge weights in each network and are entered into predictive models that assume linear relationships with behavioral data. The resultant linear equation is then applied to the test data set to predict the phenotypic data.

**Localization of predictive networks**

Predictive networks were also summarized based on length-of-connection (short- versus long-range connectivity). Euclidean distance between the centroids of each brain region in the Shen atlas was used to classify short and long-range edges. First, distance was calculated for each pair of regions as: $\sqrt{\left( x_{1}-x_{0} \right)^{2}+\left( y_{1}-y_{0} \right)^{2}+\left( z_{1}-z_{2} \right)^{2}}$, where $\left( x_{1},y_{1},z_{1} \right)$ and $\left( x_{0},y_{0},z_{0} \right)$represent the centroid for any two regions. Pair-wise distances were median-separated into short- and long-range connections.

**Behavioral Analysis and Results**

We conducted a series of repeated-measures analysis of covariance (ANCOVA) to examine the associations between irritability and task behavioral measures including accuracy, reaction time (RT), mean inhibit delay (i.e., the average duration between the initial target “go” signal and the change signal), change-signal reaction time (CSRT), and self-reported frustration ratings during the task. The change signal reaction time (CSRT) represents the speed at which one can execute the flexible response, incorporating both speed and accuracy. When the change accuracy is 50%, CSRT equals the participant’s mean reaction time on change trials minus the mean inhibit delay. Often, individual accuracy rates deviated slightly from 50%, in which case an interpolation algorithm was used to calculate CSRT. Specifically, CSRT is the “go” reaction time at the Xth percentile of go trials (where X is the participant’s accuracy on change trials), minus the participant’s mean inhibit delay. Thus, the CSRT represents an individually adjusted measure of the speed of response flexibility. Given the wide age range of the sample, we examined the effect of age on CSRT and found that age was correlated with CSRT (*r*=-.32, *p*=.009 for non-frustration blocks, *r*=-.43, *p*<.001 for frustration blocks). That is, older participants had shorter CSRT compared to younger participants. This is consistent with the stop-signal literature documenting that adults have shorter SSRT (stop-signal reaction time) than children and adolescents (6,7) and a large literature showing that response inhibition, cognitive control and cognitive flexibility improve with age (8-10).

We conducted a series of repeated-measures analysis of covariance (ANCOVA) to examine the associations between irritability and the task variables. Specifically, we conducted ARI x Block (frustration vs. non-frustration blocks) × Trial (Go vs. Change trials) ANCOVA to test whether accuracy and RT varied with irritability, block type, and trial type. In addition, we conducted ARI x Block (frustration vs. non-frustration blocks) ANCOVA to examine whether mean inhibit delay, CSRT, and self-reported frustration ratings varied with irritability and block type.

Accuracy was not significantly associated with parent-reported irritability (pARI) or child-reported irritability (cARI).

For RT, results revealed significant pARI × Block interaction (*F*_1,62_=5.34, *p*=.02, η_p_^2^=.08) and pARI × Trial interaction (*F*_1,62_=12.21, *p*=.001, η_p_^2^=.17) as well as cARI × Trial interaction (*F*_1,67_=5.30, *p*=.02, η_p_^2^=.07). Specifically, higher pARI was related to slower RT at a trend level during the frustration blocks (*r*=.22, *p*=.075) but not the non-frustration blocks (*r*=.14, *p*=.27), and higher pARI was related to slower RT during the Go trials (*r*=.29, *p*=.021) but not the Change trials (*r*=.08, *p*=.52). Follow-up analysis for the cARI × Trial interaction did not show any significant effect of cARI.

For mean inhibit delay, results revealed significant pARI × Block interaction (*F*_1,62_=6.29, *p*=.02, η_p_^2^=.09). Specifically, higher pARI was related to a greater mean inhibit delay during the frustration blocks (*r*=.23, *p*=.071) than the non-frustration blocks (*r*=.10, *p*=.43).

Neither the CSRT nor the frustration ratings were significantly associated with pARI or cARI.

**Prediction of ADHD symptoms**

To help further disentangle the relationship between irritability and ADHD at a neural level, we predicted ADHD scores when controlling for cARI (ρ=0.24, RMSE=12.9, p=0.048). For comparisons with the cARI prediction, the analysis was limited to the model with only positive features. When comparing the ADHD model to the cARI model, only a single edge overlapped between the two models, suggesting the models were largely independent of each other. At the network level, the salience, subcortical, motor, and cerebellar networks are equally prominent in the ADHD model (Figure S2).

**Prediction of trait irritability using task activation**

We used task activation during each of the five main events (i.e., correct go trials, correct change trials, rigged go trials, incorrect change trials during frustration blocks and correct change trials during non-frustration blocks) in each of the 268 nodes to predict child-reported irritability using several standard machine learning algorithms. Only the *correct change trials* during the frustration blocks showed some evidence of predictability, ρ=.19-.22 (p=.04-.09, permutation testing, 1000-iteration, one-tailed) using elastic net (alpha=.25, .50, & .75) and ρ=.20 (p=.06, permutation testing, 1000-iteration, one-tailed) using ridge regression (lambda=1; see Table S1). Across all models, predictive features were similar and included activation patterns in the lateral prefrontal cortex, insula, and fusiform (see Figure S3). Using LASSO (least absolute shrinkage and selection operator) and SVR (support vector regression), task activation did not predict child-reported irritability.

**Table S1. Prediction of child-reported trait irritability using task activation**

| **Contrast** | **SVR** | **LASSO p=0.1** | **ridge**  **(lambda=1)** | **ridge (lambda=10)** | **ridge (lambda=100, p=.1)** | **elastic net (alpha=.25)** | **elastic net (alpha=.50)** | **elastic net (alpha=.75)** |
| --- | --- | --- | --- | --- | --- | --- | --- | --- |
| **Frustrating Blocks** | | | | | | | | |
| Correct Go Trials | -0.13 | -0.36 | -0.13 | -0.2 | -0.33 | -0.31 | -0.41 | -0.37 |
| Correct Change Trials | 0.09 | 0.08 | 0.2 | 0.07 | -0.24 | 0.2 | 0.22 | 0.19 |
| Rigged Go Trials | 0.07 | -0.11 | -0.13 | -0.2 | -0.32 | -0.09 | -0.08 | -0.11 |
| Incorrect Change Trials | -0.14 | 0 | 0.12 | -0.09 | -0.31 | 0.11 | -0.01 | 0.01 |
| **Non-frustrating Blocks** | | | | | | | | |
| Correct Change Trials | 0.15 | -0.16 | 0.11 | -0.04 | -0.25 | -0.29 | -0.19 | -0.16 |


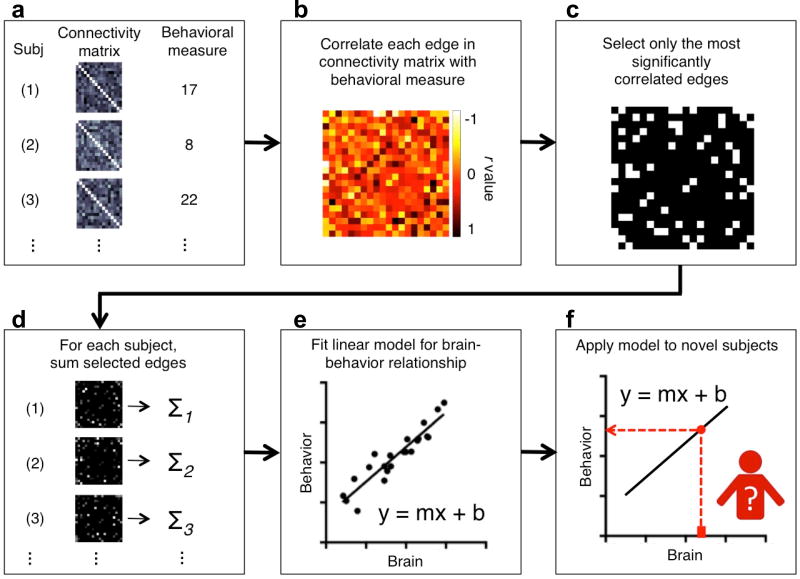


**Figure S1. Schematic of CPM.** Reproduced with permission from Shen et al. (2017) (11).


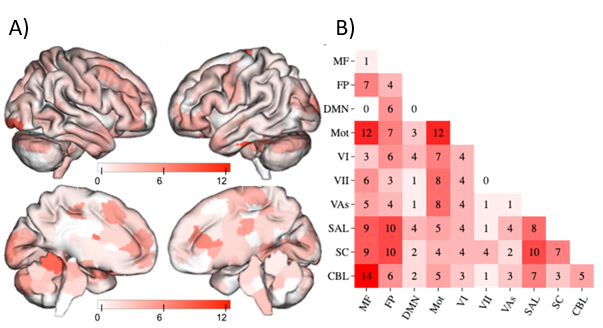


**Figure S2. ADHD model visualization.** (A) Visualization of node degree (i.e., the sum of predictive edges for a node for the positive networks). Darker color indicates higher degree. (B) Within and between network connectivity for the positive network. Cells represent the total number of edges connecting nodes within and between each network, with darker colors indicating a greater number of edges. As the negative network did not contribute to prediction, only the positive network is shown in all visualizations. Visualization created using BioImage Suite Web, <http://bisweb.yale.edu/>.

Note. MF = medial frontal; FP = frontoparietal; DMN = default mode network; Mot = motor/sensory; VI = visual A; VII = visual B; VAs = visual association; SAL = salience; SC = subcortical; CBL = cerebellum.


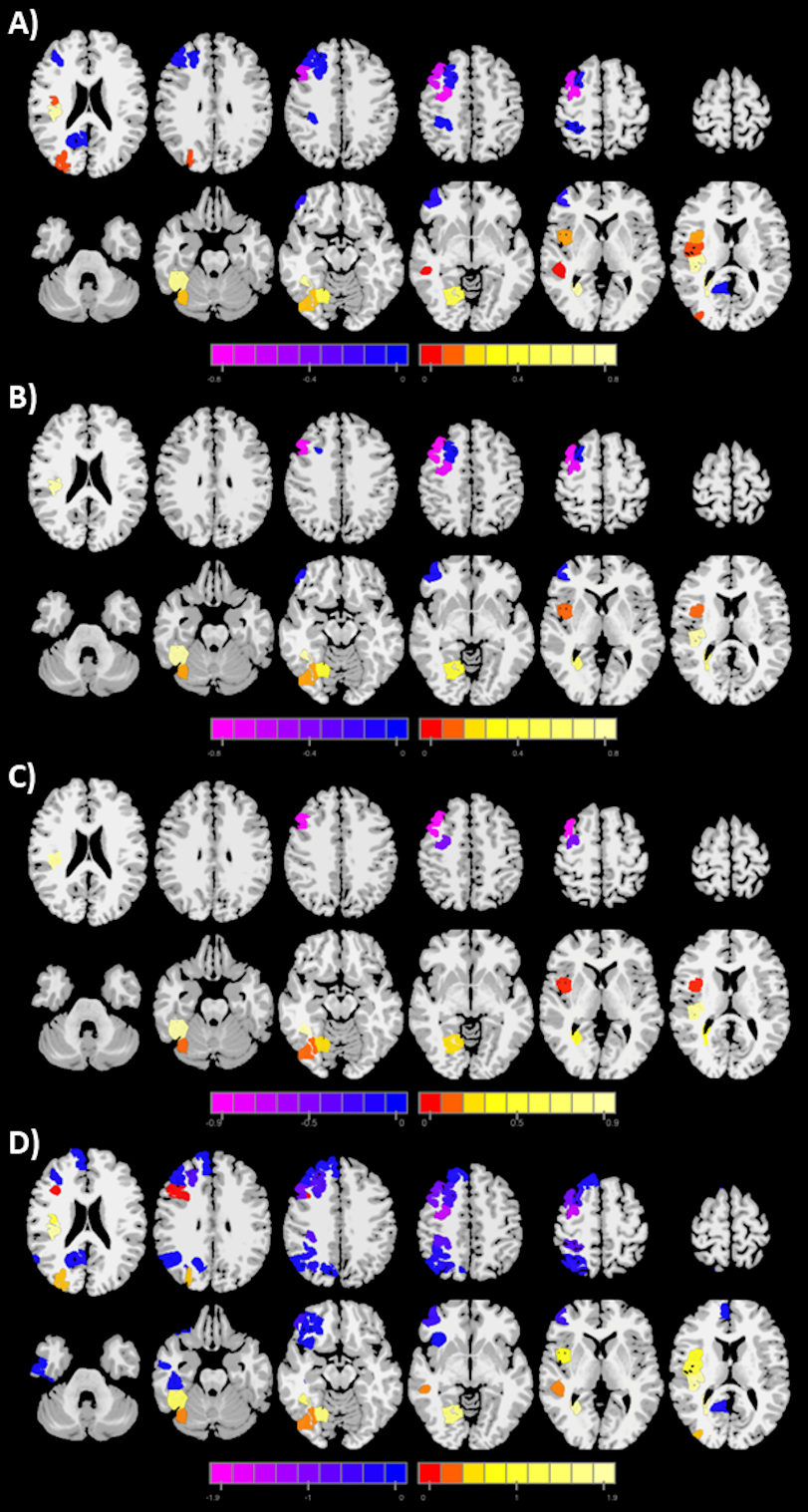


**Figure S3. Visualization of predictive features from activation-based models.** The predictive features from the activation-based models during Correct Change Trials during the frustrating blocks — A) elastic nets α=.25, B) elastic nets α=.50, C) elastic nets α=.75, D) ridge regression λ=1 — were similar and included activation patterns in lateral prefrontal cortex, insula, and fusiform. The main difference between models is the sparseness of predictive features with ridge regression being the least sparse and elastic nets with α=.75 being the most sparse.

**References**

1. Kaufman J, Birmaher B, Brent D, Rao U, Flynn C, Moreci P, et al. Schedule for Affective Disorders and Schizophrenia for School-Age Children-Present and Lifetime Version (K-SADS-PL): Initial Reliability and Validity Data. J Am Acad Child Adolesc Psychiatry. 1997 Jul 1;36(7):980–8.

2. Conners CK, Sitarenios G, Parker JD, Epstein JN. The revised Conners’ Parent Rating Scale (CPRS-R): factor structure, reliability, and criterion validity. J Abnorm Child Psychol. 1998 Aug;26(4):257–68.

3. Birmaher B, Brent DA, Chiappetta L, Bridge J, Monga S, Baugher M. Psychometric properties of the Screen for Child Anxiety Related Emotional Disorders (SCARED): a replication study. J Am Acad Child Adolesc Psychiatry. 1999 Oct;38(10):1230–6.

4. Behrens B, Swetlitz C, Pine DS, Pagliaccio D. The Screen for Child Anxiety Related Emotional Disorders (SCARED): Informant Discrepancy, Measurement Invariance, and Test-Retest Reliability. Child Psychiatry Hum Dev. 2019;50(3):473–82.

5. Brown JW, Braver TS. Learned Predictions of Error Likelihood in the Anterior Cingulate Cortex. Science. 2005 Feb 18;307(5712):1118–21.

6. Williams BR, Ponesse JS, Schachar RJ, Logan GD, Tannock R. Development of inhibitory control across the life span. Dev Psychol. 1999;35:205–13.

7. Verbruggen F, Logan GD. Response inhibition in the stop-signal paradigm. Trends Cogn Sci. 2008;12:418–24.

8. Luna B, Padmanabhan A, O’Hearn K. What has fMRI told us about the Development of Cognitive Control through Adolescence? Brain Cogn. 2010;72(1):101–13.

9. Rubia K, Smith AB, Woolley J, Nosarti C, Heyman I, Taylor E, et al. Progressive increase of frontostriatal brain activation from childhood to adulthood during event-related tasks of cognitive control. Hum Brain Mapp. 2006;27:973–93.

10. Davidson MC, Amso D, Anderson LC, Diamond A. Development of cognitive control and executive functions from 4 to 13 years: Evidence from manipulations of memory, inhibition, and task switching. Neuropsychologia. 2006;44:2037–78.

11. Shen X, Finn ES, Scheinost D, Rosenberg MD, Chun MM, Papademetris X, et al. Using connectome-based predictive modeling to predict individual behavior from brain connectivity. Nat Protoc. 2017;12(3):506–18.
